# Multifunctional Ag-decorated g-C_3_N_4_ nanosheets as recyclable SERS substrates for CV and RhB detection

**DOI:** 10.1039/c8ra02657b

**Published:** 2018-06-15

**Authors:** Yunfeng Ma, Lili Yang, Yong Yang, Yusi Peng, Yuquan Wei, Zhengren Huang

**Affiliations:** State Key Laboratory of High Performance Ceramics and Superfine Microstructure, Shanghai Institute of Ceramics, Chinese Academy of Sciences 1295 Dingxi Road Shanghai 200050 P. R. China yangyong@mail.sic.ac.cn +86-21-5241-4321; University of Chinese Academy of Sciences Beijing 100039 China

## Abstract

In this study, g-C_3_N_4_/Ag hybrid nanostructures were fabricated by facilely decorating silver nanoparticles on atmosphere-treated g-C_3_N_4_ and served as efficient SERS-active substrates. The observed significant SERS enhancement of crystal violet (CV) molecules on g-C_3_N_4_/Ag could be attributed to the high ability to concentrate target molecules through π–π stacking interactions and the near-field enhancement caused by the boosting SPR effect of the Ag NPs. The atmosphere and calcination time have a considerable impact on the SERS enhancement effect of the g-C_3_N_4_/Ag substrate. Furthermore, it took only 10 min to degrade dye molecules under visible light, and after 6 cycles the g-C_3_N_4_/Ag substrates still maintained sensitive SERS activity. This research indicates that g-C_3_N_4_/Ag hybrids can be applied as reusable SERS substrates.

## Abstract 2

In this study, g-C_3_N_4_/Ag hybrid nanostructures were fabricated by facilely decorating silver nanoparticles on atmosphere-treated g-C_3_N_4_ and served as efficient SERS-active substrates.

## Graphical abstract

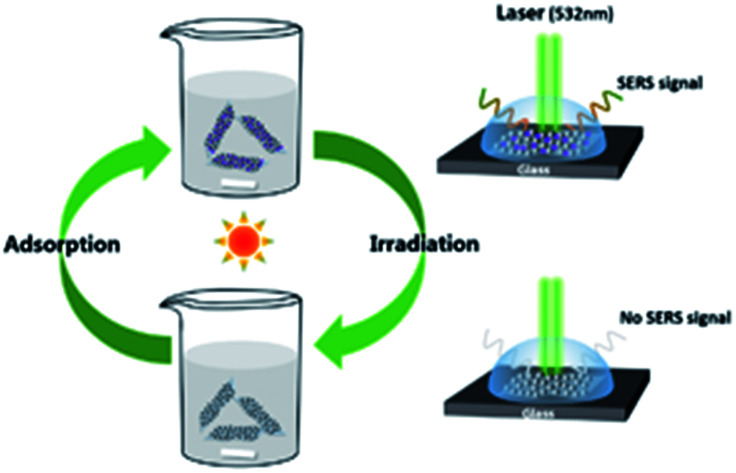

## Introduction

Surface-Enhanced Raman Spectroscopy (SERS) attracts a lot of attention since it presents a powerful tool for ultrasensitive vibrational spectroscopy, which is utilised in many frontier fields, including analytical chemistry,^1–3^ food security,^4^ life science^5^ and bio-sensing.^6^ The efficient coupling of a plasmon-induced near field with the vibrational modes of specific molecules on SERS-active substrates can enhance the Raman cross-section of the analytes by 10^4^ to 10^14^.^7–9^ When the target molecules adsorb on the proper positions of metal nanostructures, especially Au and Ag^10,11^, single molecule detection is fulfilled.^3,12–14^ It has been commonly assumed that plasma nanostructure SERS substrates can cause much greater SERS enhancement than most pure semiconductor SERS substrates,^15–18^ which originates from the “hot spots”^19^ that exist in the gaps between noble metal nanostructures. This has inspired great interest in developing highly efficient SERS-active nanostructures, including colloidal Au and Ag with various morphologies,^20–22^ nanoparticles obtained by annealing thin films,^23^ and metal coated lithography templates.^24^ However, there are still some problems; the oxidation of metal nanoparticles leads to a rapid decrease in the SERS-activity, and the fabrication procedures of coating and lithography are expensive and complicated. Moreover, noble metal substrates suffer from an obvious shortcoming, in that analytes reside on the surfaces which makes them difficult to reuse. Therefore, more research efforts have focused on preparing recyclable SERS substrates, such as TiO_2_/metal^25,26^ and ZnO/metal.^27,28^ For example, Au-coated ZnO nanorods were investigated as efficient and recyclable SERS-active substrates, with self-cleaning of the adsorbed analytes achieved through a UV degradation process.^29^ Apparently, most of the composite SERS substrates mentioned exhibit degradation activity in the UV-light region, therefore it would be meaningful and interesting to explore the preparation of a recyclable-substrate that degrades under solar-light.^30^ According to recent reports, g-C_3_N_4_ exhibits unique properties of visible light absorption and photochemical stability.^31–33^

Polymeric graphitic carbon nitride (g-C_3_N_4_) is a novel two-dimensional semiconductor material consisting of tri-*s*-triazine units, with strong covalent C–N bonds in each layer and weak van der Waals forces between layers. This material has received considerable attention owing to its splendid photoelectronic properties, extending its applications to hydrogen production,^34,35^ pollutant degradation,^36–40^ CO_2_ reduction^41,42^ and the synthesis of benzaldehyde.^43^ It is a widely held view that the prolonged carrier lifetime and improved electron transport kinetics as a result of a short diffusion path coupled with the quantum confinement effect make g-C_3_N_4_ a promising photocatalytic material. In addition, g-C_3_N_4_/metal hybrids can more effectively adsorb and enrich target molecules by π-π stacking interactions compared with noble metals,^30,44,45^ which makes the hybrids fascinating candidates for SERS applications.

Herein, we first reported g-C_3_N_4_/Ag nanocomposites with multifunctionality, whereby modified g-C_3_N_4_ was synthesized by the thermal treatment of pristine g-C_3_N_4_ at 550 °C for 1–3 hours, and then decorated with Ag NPs. The as-synthesized hybrid nanostructures not only exhibited excellent SERS activity, but also exhibited the ability to degrade RhB. After six detection/degradation cycles, g-C_3_N_4_/Ag still maintained strong SERS activity.

## Results and discussion

### Element, microstructure and optical properties characterization

**Fig. 1:**
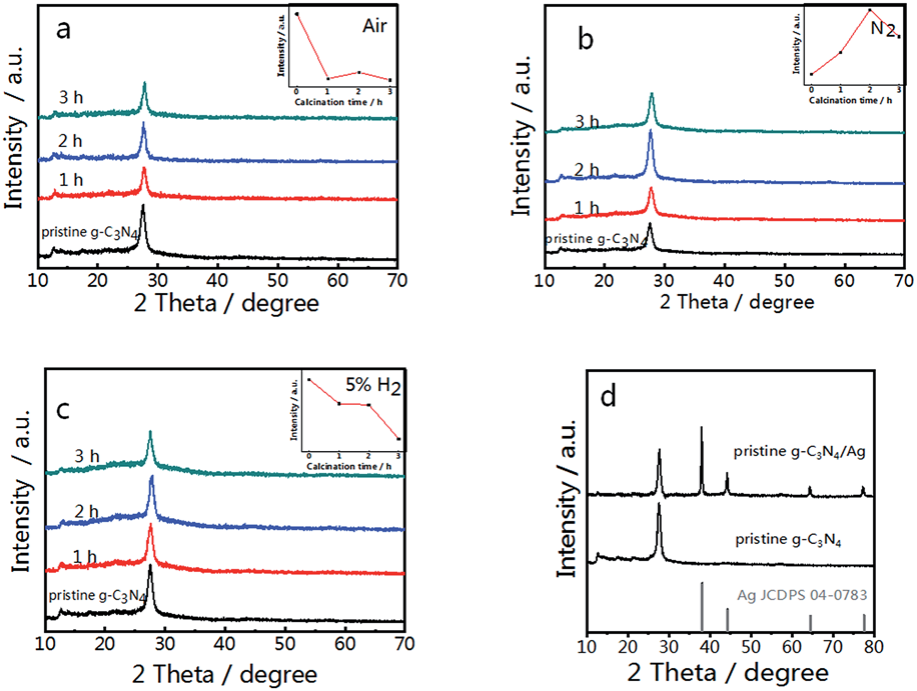
XRD patterns of g-C_3_N_4_ synthesized in different atmospheres; (a) air, (b) N_2_ and (c) 5% H_2_. (d) XRD patterns of pristine g-C_3_N_4_ and the pristine g-C_3_N_4_/Ag composite. The insets of (a)–(c) show the relationship between the peak intensity at 27.5° and the calcination time.

Pristine g-C_3_N_4_ comprised of tri-*s*-triazine units was reported to form from the thermal condensation of nitrogen-rich compounds under an air and nitrogen atmosphere.^34,46^ For comparison, treated g-C_3_N_4_ was synthesized under different atmospheres (air, N_2_, and 5% H_2_ + 95% N_2_) at the same temperature and calcination time. These samples were characterized by X-ray diffraction (XRD), and all samples demonstrated a typical graphite-like C_3_N_4_ structure with two distinctive peaks at 12.8° and 27.5°.^34^ The peak at 12.8° corresponds to the in-plane structure of the tri-*s*-triazine units while the peak at 27.5° is attributed to the interplanar stacking similar to that observed in the graphite layer structure. The insets of Fig. 1(a–c) show the relationship between the peak intensity at 27.5° and the calcination time. As shown in Fig. 1(a), the diffraction intensity decreases dramatically with increased time, corresponding to the oxide-peeling of g-C_3_N_4_.^37^ However, the diffraction intensity of the N_2_ treated g-C_3_N_4_ reaches its highest (Fig. 1(b)) after 2 h of heating in N_2_, illustrating the interlaminar stacking of g-C_3_N_4_. Fig. 1(c) shows that the diffraction intensity of 5% H_2_ treated g-C_3_N_4_ decreases with calcination time, and the diffraction peak shifts to a higher diffraction angle, thus suggesting that hydrogen elements were inserted into the g-C_3_N_4_ lattice and bulk doping was achieved. Furthermore, the characteristic peak of g-C_3_N_4_ and the four characteristic peaks of Ag such as the (111), (200), (220) and (311) planes can be observed in Fig. 1(d) in the XRD pattern of g-C_3_N_4_/Ag.

**Fig. 2:**
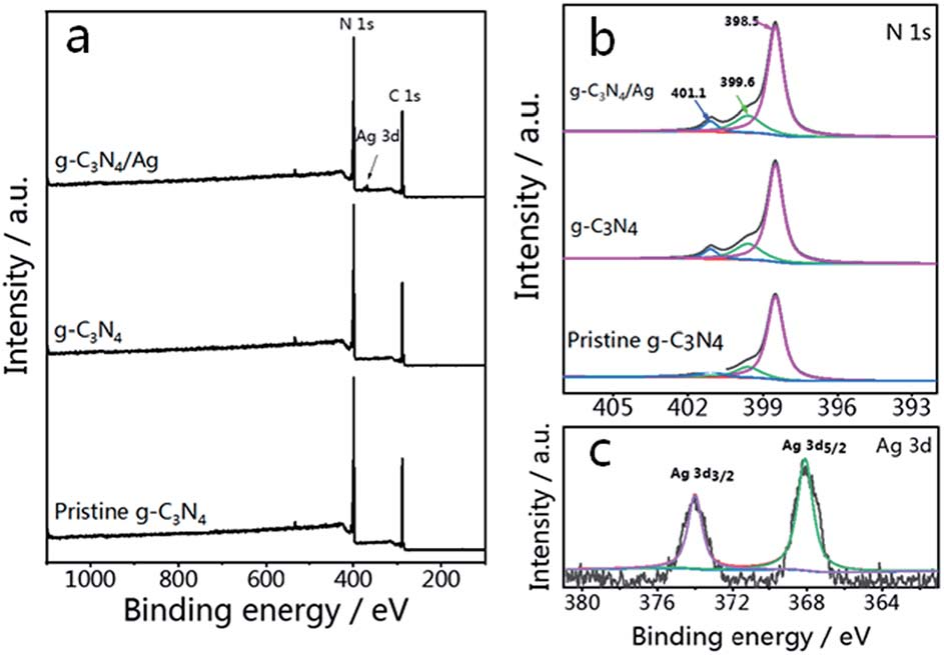
(a) XPS survey scans and (b) high resolution N 1s spectra of pristine g-C_3_N_4_, g-C_3_N_4_ and g-C_3_N_4_/Ag. (c) Ag 3d spectra of g-C_3_N_4_/Ag.

The survey spectrum and high resolution XPS spectra of various elements in pristine g-C_3_N_4_, g-C_3_N_4_ and g-C_3_N_4_/Ag are shown in Fig. 2(a). There are C, N and O elements in pristine g-C_3_N_4_ and g-C_3_N_4_, as can be seen in the survey spectrum, while peaks corresponding to C, N, O and Ag signals appear in the spectrum of g-C_3_N_4_/Ag. It should be pointed out that the peak seen at 532.1 eV in the survey scan is ascribed to the adsorbed H_2_O.^47^ It is noteworthy that the specific peaks of C 1s and N 1s in the corresponding high resolution spectra of pristine g-C_3_N_4_, g-C_3_N_4_ and g-C_3_N_4_/Ag are basically stable, while the relative amounts of C and N show a certain change. The C/N atomic ratios of the pristine g-C_3_N_4_, g-C_3_N_4_ and g-C_3_N_4_/Ag are 0.76, 0.81 and 0.82, all of which are close to the theoretical value of 0.75 (element ratio of C_3_N_4_). In the high resolution XPS spectra of N 1s in Fig. 2(b), the peaks at 398.5 eV can be ascribed to C–N=C coordination, which originates from the sp^2^-bonded N in the tri-*s*-triazine units, and the other two weak peaks at higher binding energies (around 399.6 and 401.1 eV) can be attributed to the N–(C)_3_ and C–N–H groups, respectively.^48^ Interestingly, the percentage of the peak at 399.6 eV increases from 15.8% to 20.6%, whereas the percentage of the peak located at 401.1 eV decreases from 6.9% to 6.5% for pristine g-C_3_N_4_ compared with N_2_ treated g-C_3_N_4_, which demonstrated that more carbon replaced H atoms. The change of the C/N ratio is consistent with the elementary analysis results previously measured. After loading Ag NPs, the ratio of N(sp^2^)/N(sp^3^) increases from 3.53 to 3.69. The result of the interaction between Ag and g-C_3_N_4_ is that some sp^3^-hybridized nitrogen transforms into sp^2^-hybridized nitrogen.^45^Fig. 2(c) shows two peaks, located at 368.1 and 374.0 eV with a splitting of 5.9 eV, representing the metallic Ag 3d_5/2_ and Ag 3d_3/2_ binding energies.^49^

**Fig. 3:**
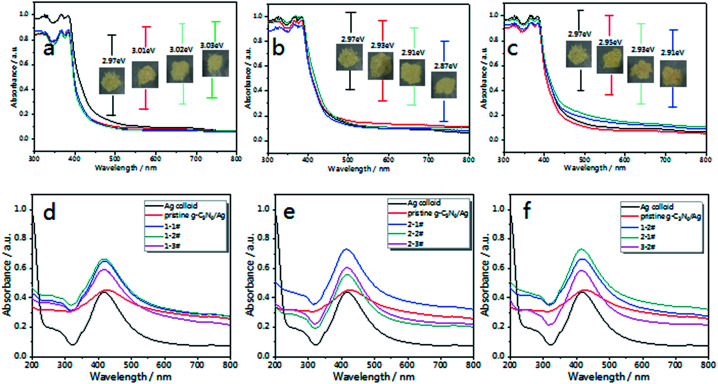
UV-visible diffuse reflectance spectra of the as-prepared g-C_3_N_4_ in different atmospheres; (a) air, (b) N_2_ and (c) 5% H_2_. The inset images show the picture and band gap of each sample. After being decorated with Ag NPs, the UV-visible absorption spectra of (d) g-C_3_N_4_/Ag composites treated in air (named 1-1#, 1-2# and 1-3#) and (e) g-C_3_N_4_/Ag composites treated in N_2_ (named 2-1#, 2-2# and 2-3#). (f) UV-vis absorption spectra of g-C_3_N_4_/Ag (1-2#, 2-1# and 3-2#).

The optical properties of g-C_3_N_4_ were measured *via* UV-vis DRS (Diffuse Reflectance Spectra). Fig. 3(a–c) shows the UV-vis DRS of the as-prepared g-C_3_N_4_ in different atmospheres. The inset pictures show the band gap (*E*_g_) of each sample. Compared with pristine g-C_3_N_4_, the optical absorption intensity of air treated g-C_3_N_4_ decreases with calcination time in the visible region, which is caused by oxide-stripping, while the N_2_ and 5% H_2_ treated g-C_3_N_4_ samples exhibit increased absorption in the visible region.

The optical band gap (*E*_g_) of g-C_3_N_4_ can be deduced according to the following equation:(*αhν*)^*n*^ = *A*(*hν* − *E*_g_)^50^where *α* is the absorption coefficient, *hν* is the incident photon energy, the value of the index *n* we chose was *n*_direct_ = 2,^46^*A* is a proportionality constant related to the material, and *E*_g_ is the band gap energy of the semiconductor. As shown in Fig. 3(a–c), the *E*_g_ of pristine g-C_3_N_4_ was obtained, which was around 2.97 eV^51^ corresponding to the absorption edge that appeared at about 420 nm.

After being decorated with Ag NPs, the UV-vis absorption spectra of the g-C_3_N_4_/Ag composites were measured, with the concentration of Ag (for both the Ag colloid and the g-C_3_N_4_/Ag composites) tuned to 0.067 mM. As shown in Fig. 3(d–f), Ag NPs exhibit a strong plasma absorption band at 420 nm, which is caused by the collective oscillation of electrons on the surfaces of the Ag NPs.^52,53^ After being combined with pristine g-C_3_N_4_, the composites exhibit an increased absorption over the whole visible region and the absorption peak gradually red-shifts to 430 nm, indicating the electronic interactions between the Ag NPs and g-C_3_N_4_ nanosheets, and exhibiting the aggregation state of the Ag NPs on pristine g-C_3_N_4_.^54^ Therefore a variation in the dielectric surroundings could greatly change the LSPR behavior of the Ag NPs.

**Fig. 4:**
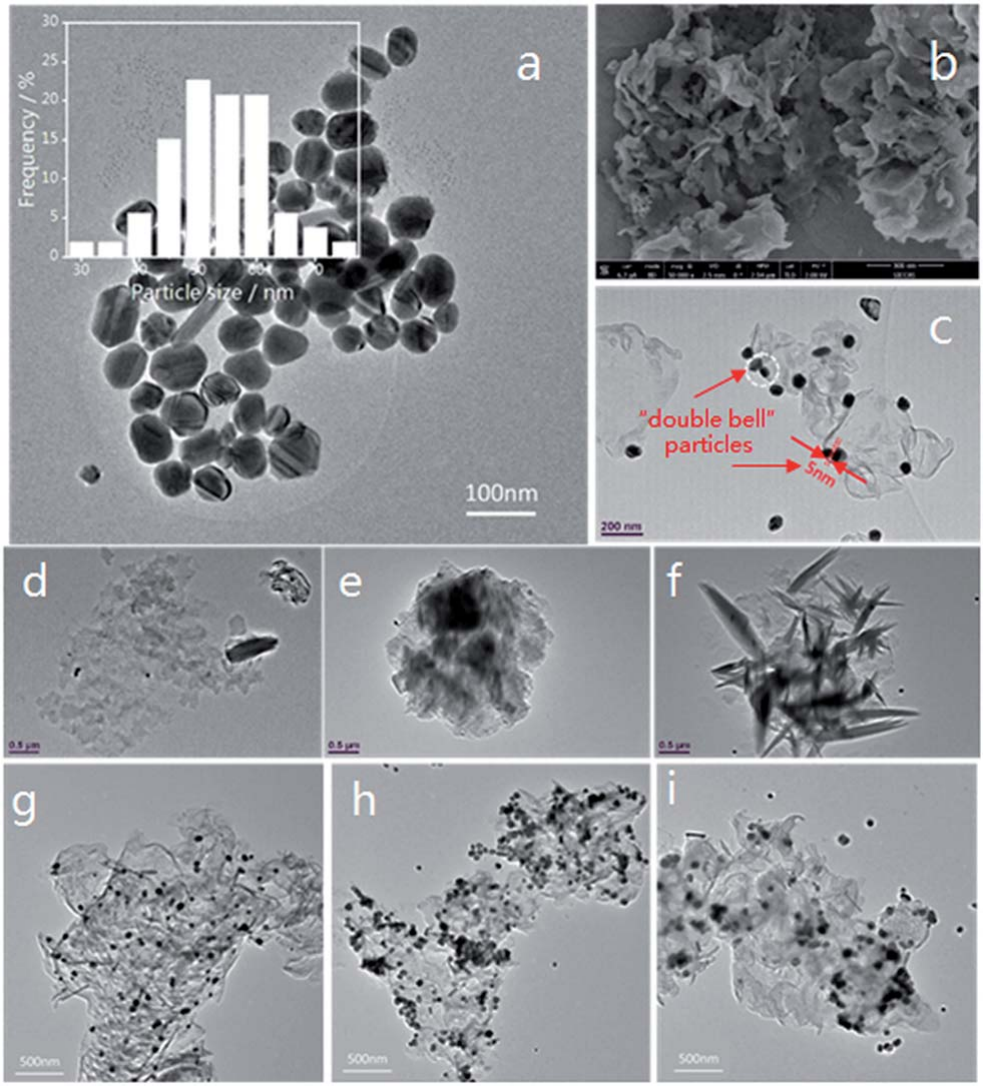
(a) A TEM image of silver nanoparticles and the corresponding size distribution, scale bar = 100 nm. (b) SEM image of pristine g-C_3_N_4_, scale bar = 500 nm. (c) TEM image of Ag nanoparticle decorated pristine g-C_3_N_4_, scale bar = 200 nm. (d–f) TEM images of g-C_3_N_4_ (N_2_ treated), scale bar = 500 nm. (g–i) TEM images of g-C_3_N_4_/Ag (1-2# (air-2 h), 2-1# (N_2_-1 h) and 3-2# (5% H_2_-2 h)), scale bar = 500 nm.

Furthermore, TEM and SEM characterization was carried out, and it can be clearly seen from the inset histogram graph of Fig. 4(a) that 82% nanoparticles have particle sizes of between 45–60 nm, and that the average diameter of the Ag NPs is 50 nm, indicating that the size distribution of the silver nanoparticles is relatively narrow and uniform. Fig. 4(b) shows that pristine g-C_3_N_4_ is a porous framework comprised of a two-dimensional layered structure and the edge of g-C_3_N_4_ curled up after the alcohol was volatilized completely. In Fig. 4(c) we can observe Ag NPs attached to pristine g-C_3_N_4_, with some of them forming “double bell” particles with a gap between them. The Ag NPs are easily attached to the g-C_3_N_4_ at the numerous binding sites. Furthermore, the driving force for adsorbing Ag NPs on the surface of g-C_3_N_4_ can be attributed to the coordination between the unoccupied orbitals of the Ag NPs and the lone-pair electrons of the N atoms.^55^ Moreover, the porous framework could effectively immobilize the Ag NPs. “Double bell” particles with or without a slight gap could form an effective “hot spot”^19,56^ originating from the electric field coupling near to the Ag NPs, which will contribute to the SERS effect.

Fig. 3(d and e) illustrate the changes in the absorption spectra of the g-C_3_N_4_/Ag samples. Compared with the pristine g-C_3_N_4_/Ag composites, there is no noticeable change in the width and position of the SPR band, which implies there is no excessive agglomeration. According to our knowledge, a strong local electric field could be produced around the Ag NPs under the action of external electric field. When g-C_3_N_4_ is in contact with the Ag NPs, a quasi-fermi level will form in the g-C_3_N_4_/Ag nanocomposites. The photo-excited electrons could transfer from g-C_3_N_4_ to the surface of the Ag NPs and change the distribution state of the surface charge around the Ag NPs,^53^ thus affecting the energy state of plasma, and eventually leading to the difference in the UV-vis spectra that we observed. Fig. 3(f) shows the absorption spectra of g-C_3_N_4_/Ag (1-2#, 2-1# and 3-2#) treated in different atmospheres. The g-C_3_N_4_/Ag composite treated in N_2_ for 1 h (2-1#) exhibited the strongest absorption intensity. We predict that an effective promotion of the absorption intensity will improve the optical properties of the g-C_3_N_4_/Ag composites. It is worth noting that the “hot spot” distribution and charge transfer process from the substrate to the adsorbed analytes correspond to two main SERS enhancement mechanisms (EM and CT).^57^ Therefore, these g-C_3_N_4_/Ag composites possessing photo-excited features must be extraordinary SERS materials.

The morphology of the N_2_ treated g-C_3_N_4_ was analysed by TEM (Fig. 4(d–f)). The size of the g-C_3_N_4_ nanosheets is on the micron scale, and the thickness of g-C_3_N_4_ is about 2 nm according to the AFM results. Consistent with the XRD analysis results, with increased calcination time the g-C_3_N_4_ became flatter. However, after 2–3 h of heating, the g-C_3_N_4_ went through partial re-aggregation and was then damaged by thermal effects,^58^ as Fig. 4(f) demonstrates.

The loading of the Ag NPs onto g-C_3_N_4_ was also characterized by TEM, and typical images of the 1-2#, 2-1# and 3-2# samples at the same magnification are shown in Fig. 4(g–i). The distribution of Ag NPs on g-C_3_N_4_ (air-2 h) is relatively uniform, with adjacent particles occupying a large proportion of the Ag NPs. The amount of Ag NPs on g-C_3_N_4_ (N_2_-1 h) is the largest, and we find that there are some overlapping areas on the sample. However, for the g-C_3_N_4_/Ag (5% H_2_-2 h) sample, the Ag NPs were not effectively adsorbed on the g-C_3_N_4_. The distribution of “hot spots” on these substrates may cause the difference in the SERS performance that we will discuss below.

### Multifunctional Ag decorated g-C_3_N_4_ nanosheets: SERS substrate and catalytic properties

**Fig. 5:**
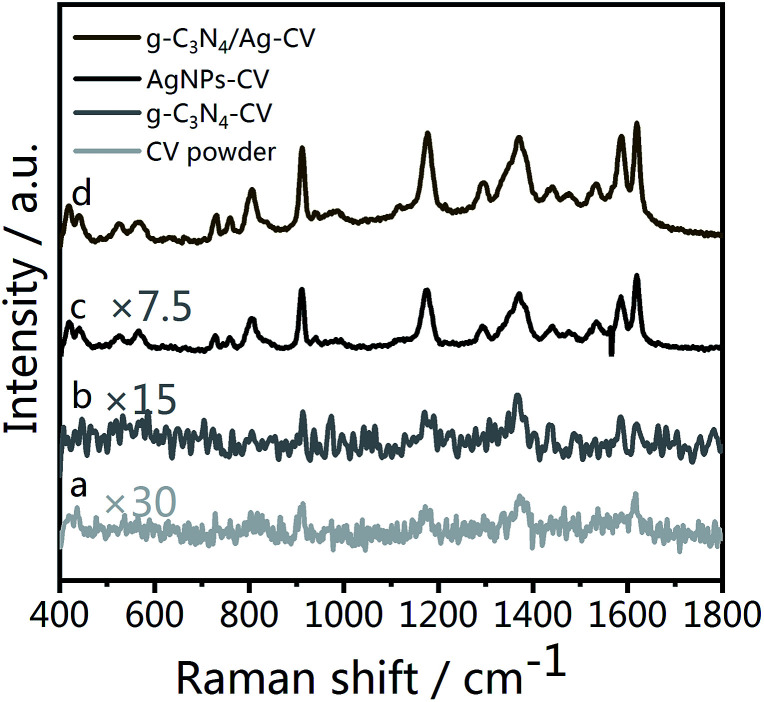
(a) Raman spectrum of CV powder, and SERS spectra of CV on (b) g-C_3_N_4_, (c) Ag NPs and (d) g-C_3_N_4_/Ag.

As Fig. 5 illustrates, fingerprint bands in the spectra of CV powder (Fig. 5(a)), corresponding to aromatic C–C stretching modes at 1620, 1533 and 1442 cm^−1^, an *N*-phenyl stretching mode at 1370 cm^−1^, and aromatic C–H bending modes at 1179, 912 and 806 cm^−1^, are observed, which agree well with literature data.^59^Fig. 5(d) shows the Raman spectrum of CV molecules adsorbed on the g-C_3_N_4_/Ag nanostructure at a laser excitation wavelength of 532 nm. For comparison, unbound Ag NPs (Fig. 5(c)) were used as a reference sample. The SERS signal of CV on pristine g-C_3_N_4_ is weak (Fig. 5(c)), whereas the peaks at 1620, 1588, 1370, 1179, 912 and 806 cm^−1^ can still be distinguished. The π–π stacking interactions between g-C_3_N_4_ and CV could change the distribution of charge density of the CV molecules, which specifically leads to the enhancement of several characteristic peaks. This is the feature of chemical enhancement.^60^ The SERS signal of CV molecules from g-C_3_N_4_/Ag hybrids is at least one order of magnitude higher than that from Ag NPs alone, indicating the excellent SERS activity of the as prepared g-C_3_N_4_/Ag hybrids. This observed phenomenon could be explained by the presence of “hot spots” attributed to its plasmonic near-field enhancement structure.^19^ Therefore, the SERS enhancement mechanism of this hybrid substrate can be attributed to the combined action of electromagnetic enhancement and chemical enhancement caused by charge transfer.

Due to the complicated distribution of the g-C_3_N_4_/Ag composites, it is not easy to accurately compute the enhancement factor (EF). Therefore, we obtained at least 10 spectra and calculated an average for each sample. The EF is calculated by the following equation:EF = (*I*_SERS_/*I*_Raman_)(*C*_Raman_/*C*_SERS_)^9^where *I*_SERS_ and *I*_Raman_ are the integrated intensities of the SERS signal (1620 cm^−1^) and normal Raman spectra, respectively. *C*_SERS_ and *C*_Raman_ are the concentrations of molecules adsorbed on the g-C_3_N_4_/Ag hybrids and in the solution sample (0.01 M). In this experiment, the diameter of the laser beam spot was 1 μm.

**Fig. 6:**
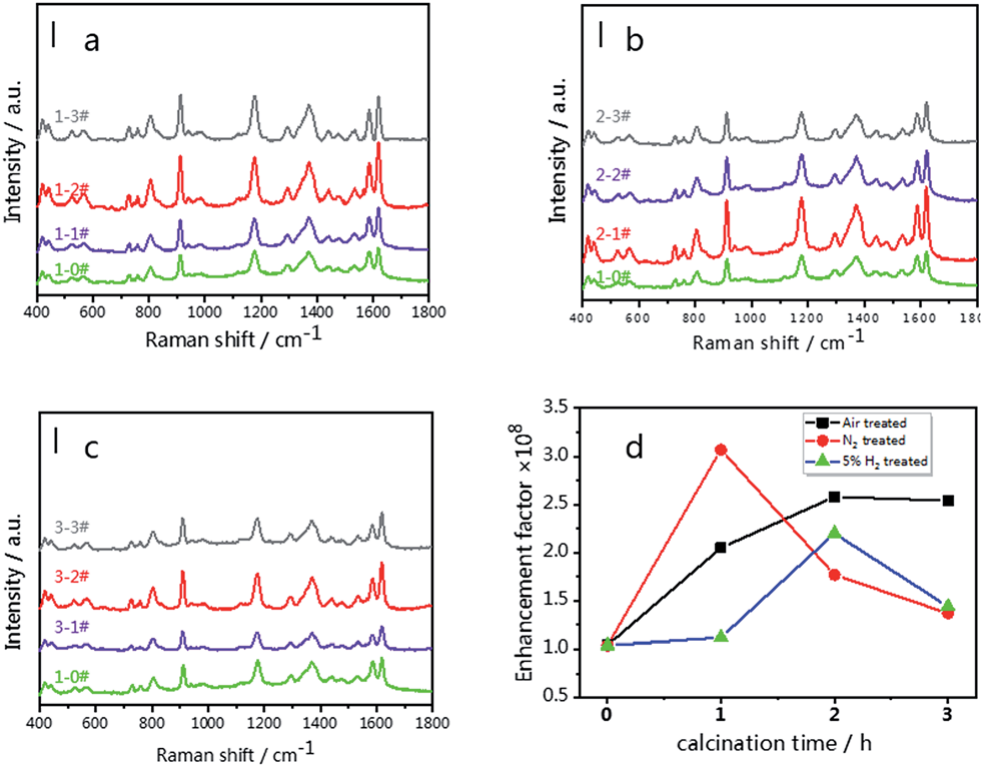
SERS spectra of 10^−6^ M CV on the g-C_3_N_4_/Ag SERS substrates; (a) g-C_3_N_4_/Ag substrates treated in air (1-1#, 1-2# and 1-3#), (b) g-C_3_N_4_/Ag substrates treated in N_2_ (2-1#, 2-2# and 2-3#) and (c) g-C_3_N_4_/Ag substrates treated in 5% H_2_ (3-1#, 3-2#, 3-3#). (d) Enhancement factor tendency of the g-C_3_N_4_/Ag SERS substrates (air, N_2_ and 5% H_2_). The green lines in Fig. 6(a–c) represent the spectrum of 10^−6^ M CV on the pristine-g-C_3_N_4_/Ag SERS substrate. Scale bar = 10 000 cps. A 532 nm laser was used at 0.5 mW with an integration time of 2 s × 2 times per spectrum.

Fig. 6(a–c) illustrate the SERS spectra of 10^−6^ M CV on the g-C_3_N_4_/Ag substrates treated in different atmospheres. Upon increasing the treatment time, the integral strength at 1620 cm^−1^ shows a tendency to increase at first and subsequently decrease. Optimum results are obtained for the 1-2# (air-2 h), 2-1# (N_2_-1 h) and 3-2# (5% H_2_-2 h) samples, respectively. Among them, the 2-1# (N_2_-1 h) sample exhibits the highest SERS signal (Fig. 6(d)), which is 30 times higher than that of the individual Ag NPs SERS platform as demonstrated by the green line shown in Fig. 6(a–c). This phenomenon also confirms that the excellent visible light absorption properties and higher “hot spot” density result in a stronger near-field enhancement in the g-C_3_N_4_/Ag system.

Over the past few decades, semiconductors and composite materials have been widely used for the removal of environmentally hazardous compounds. Now researchers are looking into the method of reusing composite SERS substrates by the photocatalytic degradation method. We employed our as-synthesized g-C_3_N_4_ (N_2_-1 h)/Ag composites for the degradation of rhodamine B (RhB) under sunlight irradiation.

**Fig. 7:**
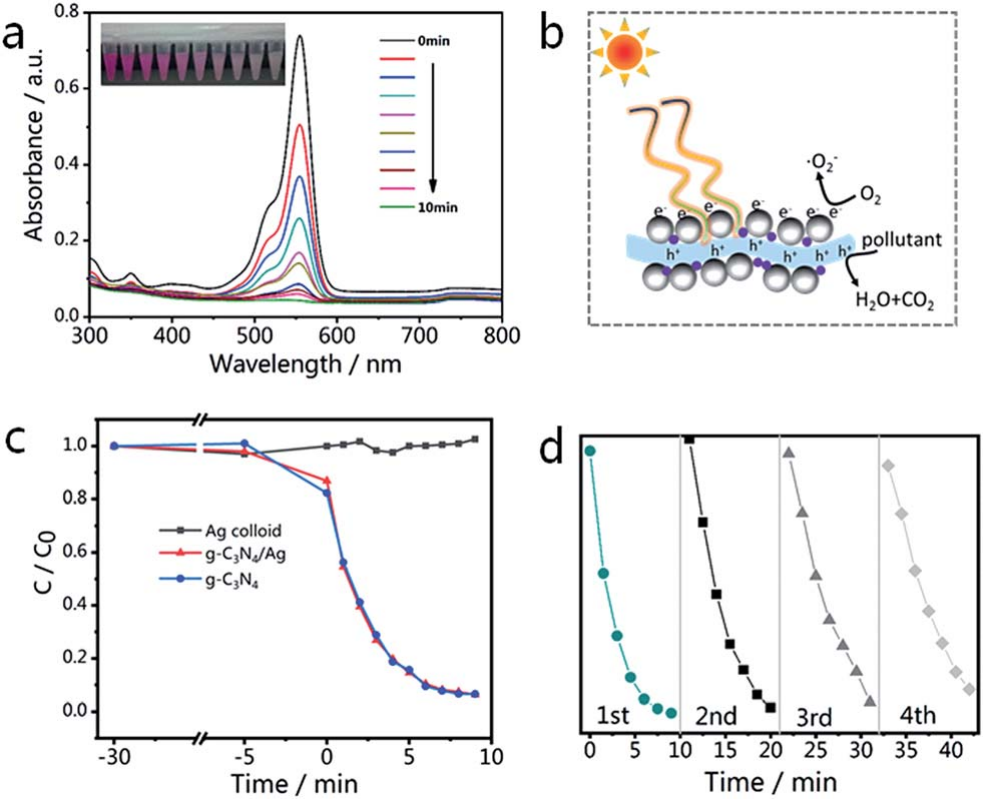
(a) UV-vis spectra of RhB with g-C_3_N_4_/Ag composites after illumination by visible light for 10 min; the inset photograph shows the actual color change of RhB. (b) Electron transfer mechanism for the photocatalytic degradation of an organic pollutant using g-C_3_N_4_/Ag composites under visible light irradiation. (c) The photocatalytic activities of Ag colloid, g-C_3_N_4_/Ag and g-C_3_N_4_ towards RhB degradation. (d) Recycling properties of the photocatalytic degradation of RhB over g-C_3_N_4_/Ag in the visible region.

The changes in the UV-vis spectra during photodegradation are shown in Fig. 7(a), with the main absorbance peak of RhB at 553 nm decreasing rapidly with increased irradiation time. The color of the sample fades almost completely after 10 min, while the aforementioned peak also becomes invisible. There is no obvious blue-shift of the absorbance peak at 553 nm (corresponding to N-demethylation), which suggests that the degradation of RhB occurs mainly *via* the destruction of the conjugated structure.^61^ The blank experiment result (Fig. 7(c)) indicates that the degradation of RhB can be neglected in the absence of catalysts (Ag NPs only). Looking at the results shown in Fig. 7(c), it is not difficult to see that the photocatalytic activity of the g-C_3_N_4_/Ag composite is very close to that of g-C_3_N_4_. The degradation rates of RhB over the g-C_3_N_4_/Ag and g-C_3_N samples are 93.7 and 93.4% after 9 min, respectively. The photodegradation stability of the as-synthesized g-C_3_N_4_/Ag composite was determined by conducting a recycling experiment. As shown in Fig. 7(d), the mixed solution was quickly decolorized after each photodegradation process and the photocatalytic activity of the g-C_3_N_4_/Ag nanocomposites only slightly decreased after four cycles.

**Fig. 8:**
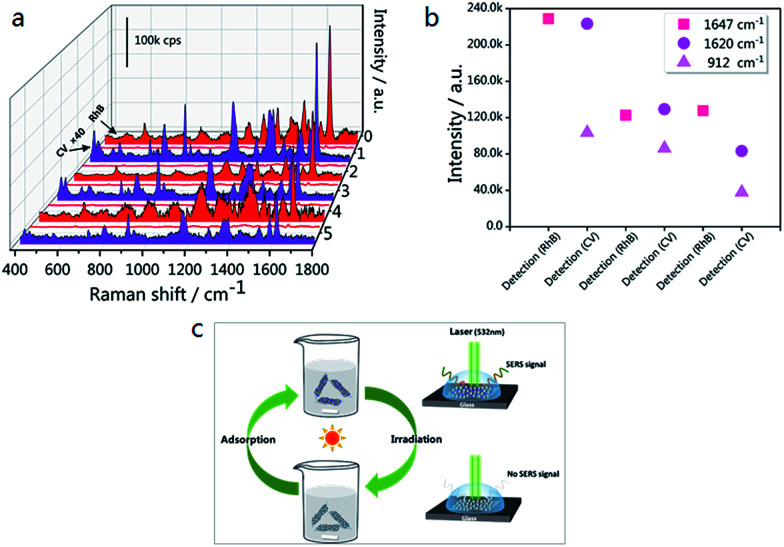
(a) SERS spectra of six detection/degradation cycles of RhB (10^−5^ M) and CV (10^−6^ M); each cycle consists of the adsorption of target molecules followed by visible light irradiation. The acquisition parameters for RhB detection were 2 accumulations at 10 s exposure, while those for CV detection were 2 accumulations at 2 s exposure. The laser power that was used was 0.025 mW. The Raman intensity of CV was multiplied by a factor of 40. (b) The Raman intensity changes of RhB at 1647 cm^−1^, and CV at 912 and 1620 cm^−1^. (c) A schematic diagram demonstrating the recyclable use of the g-C_3_N_4_/Ag substrates.

In order to expand the applications of the g-C_3_N_4_/Ag hybrids, SERS measurements were carried out, with the results shown in Fig. 8(a). In the first cycle, the SERS signal of RhB was unobserved after 10 min of visible light irradiation. After the degradation of RhB, CV and RhB were alternately used as probe molecules for detection. Six detection and degradation cycles later, the SERS intensity of RhB and CV on g-C_3_N_4_/Ag decreases, but still remains at a certain level. Fig. 8(b) illustrates the fingerprints of RhB (at 1647 cm^−1^) and CV (at 1620 and 912 cm^−1^), and the two Raman characteristic peaks of CV are maintained at 37.2% and 36.7% of their original intensities, respectively. This proves that the photocatalytic activity of the g-C_3_N_4_/Ag hybrids can be developed in order to remove the adsorbed pollutants and make the substrates reusable. Fig. 8(c) displays a schematic diagram demonstrating the recyclable use of the g-C_3_N_4_/Ag substrates. Analytes with a benzene ring were gathered easily through π–π stacking interactions, and the SERS signal of the probe molecule could be detected when it adsorbed at the active site. After 10 min of visible light irradiation, no SERS signal of the target molecule was observed. The substrates were then washed twice with deionized water so that it could re-adsorb and detect probe molecules. Through the above experiments, we have found a fast and efficient way to utilize the g-C_3_N_4_/Ag SERS substrate.

## Experimental

### Chemicals

Silver nitrate (AgNO_3_), sodium citrate (Na_3_C_6_H_5_O_7_·2H_2_O), urea (>99%) and crystal violet (C_25_H_30_ClN_3_) were purchased from Aladdin Co., Ltd. All reagents were analytically pure and were used without further purification.

### Sample preparation

#### Preparation of g-C_3_N_4_

Pristine g-C_3_N_4_ was prepared by the thermal condensation of 100 g of urea at 550 °C in an alumina crucible with a cover for 4 h in static air with a ramp rate of 5 °C min^−1^. The furnace was then cooled naturally to room temperature. The resulting yellow product was collected and ground into a powder. The pristine g-C_3_N_4_ was then treated in different atmospheres (air, N_2_ and 5% H_2_) at 550 °C for 1 h, 2 h and 3 h, with the resultant samples denoted as air-1 h/2 h/3 h-g-C_3_N_4_ (1-1#, 1-2# and 1-3#), N_2_-1 h/2 h/3 h-g-C_3_N_4_ (2-1#, 2-2# and 3-3#), and 5% H_2_-1 h/2 h/3 h-g-C_3_N_4_ (3-1#, 3-2# and 3-3#).

#### Preparation of the Ag colloid

The Ag colloid was synthesized by the Lee-Meisel method.^62^ AgNO_3_ (72 mg) was dissolved in 400 mL of H_2_O, which was then brought to the boiling point. A solution of 1% sodium citrate (8 mL) was then added. The solution was continuously boiled for *ca.* 1 h.

#### Preparation of the g-C_3_N_4_/Ag composites

The g-C_3_N_4_/Ag composites were prepared by mixing 25 mg of g-C_3_N_4_ and 25 mL of the Ag colloid in a beaker. The suspension was stirred for 1 h, followed by centrifuging 25 mL of the composite solution at 10 000 rpm for 10 min, then redistributing in 25 mL deionized water for use.

50 μL of the as-prepared g-C_3_N_4_/Ag substrate suspension was dispersed in 1 mL of CV (10^−6^ M) solution, and the distributed solution was left to stand for 1 h in order to make the system uniform and reach an adsorption equilibrium before the Raman tests.

Photocatalytic experiments were carried out on the g-C_3_N_4_/Ag composites in order to recycle the substrate, with the following steps carried out: 1 mL of a 10^−3^ M RhB solution and 0.5 mL of 1 M hydrochloric acid were injected into 25 mL of the g-C_3_N_4_/Ag suspension, then stirred for 30 min under dark conditions. The purple solution was irradiated with a high xenon lamp for 10 min, and was stirred using a magnetic stirrer and maintained at 25 °C using circulating water. The irradiated g-C_3_N_4_/Ag solution was then centrifuged, washed repeatedly, dispersed in water and reused as a SERS substrate.

### Sample characterization

The crystal structures of the g-C_3_N_4_ and g-C_3_N_4_/Ag samples were determined by X-ray diffraction (XRD) using a Bruker D8 Advance X-ray powder diffractometer with a Cu Kα radiation source. The morphologies of the samples were measured using a JEOL JEM-2100F field emission source transmission electron microscope (TEM) operated at an acceleration voltage of 200 kV. The thickness of the sample was examined using a commercial atomic force microscope (AFM, NT-MDT). X-ray photoelectron spectroscopy (XPS) signals were collected using a Leybold MAX 200 photoelectron spectrometer equipped with a Mg Kα radiation source (1253.6 eV) operated at 200 W. UV-vis diffuse reflectance spectra were recorded using a PE Lambda 950 instrument. Raman spectra were obtained using a Renishaw inVia Raman spectrometer equipped with a 532 nm solid-state laser (*λ* = 532 nm). The Raman spectrum of a silicon wafer at 520.7 cm^−1^ was used to calibrate the spectrometer. Measurements of each sample were repeated 5–10 times in order to verify the reproducibility of the spectra.

## Conclusions

In conclusion, we report the synthesis of a novel g-C_3_N_4_/Ag SERS substrate by facilely decorating Ag NPs on modified g-C_3_N_4_. These g-C_3_N_4_/Ag substrates exhibit excellent SERS activity towards CV dye molecules, with the strongest enhancement factor reaching 3.0 × 10^8^. These g-C_3_N_4_/Ag hybrids exhibit outstanding performance due to the concentration of probe molecules through π–π stacking interactions. Furthermore, photo-excited electrons from the g-C_3_N_4_ are transferred to the Ag NPs, which greatly boosts the SPR effect of the Ag NPs. Therefore, the SERS enhancement mechanism of the g-C_3_N_4_/Ag hybrid substrates can be attributed to the combination of electromagnetic enhancement and chemical enhancement. Excellent SERS reusability of the g-C_3_N_4_/Ag composites is observed. The self-cleaning and reusable SERS properties of the composites make the g-C_3_N_4_/metal system a promising SERS-active substrate for use in pesticide residue detection and sewage treatment areas.

## Conflicts of interest

The authors declare no conflict of interest.

## Supplementary Material
